# Artificial intelligence in fixed implant prosthodontics: a retrospective study of 106 implant-supported monolithic zirconia crowns inserted in the posterior jaws of 90 patients

**DOI:** 10.1186/s12903-020-1062-4

**Published:** 2020-03-19

**Authors:** Henriette Lerner, Jaafar Mouhyi, Oleg Admakin, Francesco Mangano

**Affiliations:** 1 Private Practice, Ludwing-Wilhelm Strasse, 17 Baden-Baden, Germany; 2 Lecturer, Academic Teaching and Research Institution of Johann Wolfgang Goethe-University, Frankfurt am Main, Germany; 3 Casablanca Oral Rehabilitation Training & Education Center (CORTEC), Casablanca, Morocco; 4 Biomaterials Research Department, International University of Agadir (Universiapolis), Agadir, Morocco; 5 Department of Prevention and Communal Dentistry, Sechenov First Moscow State Medical University, 119992 Moscow, Russia; 6 Lecturer, Department of Prevention and Communal Dentistry, Sechenov First Moscow State Medical University, Moscow, Russia

**Keywords:** Artificial intelligence, Monolithic zirconia crowns, Individual hybrid abutments, Full digital workflow, Marginal adaptation, Survival, Success

## Abstract

**Background:**

Artificial intelligence (AI) is a branch of computer science concerned with building smart software or machines capable of performing tasks that typically require human intelligence. We present a protocol for the use of AI to fabricate implant-supported monolithic zirconia crowns (MZCs) cemented on customized hybrid abutments.

**Methods:**

The study protocol consisted of: (1) intraoral scan of the implant position; (2) design of the individual abutment and temporary crown using computer-aided design (CAD) software; (3) milling of the zirconia abutment and the temporary polymethyl-methacrylate (PMMA) crown, with extraoral cementation of the zirconia abutment on the relative titanium bonding base, to generate an individual hybrid abutment; (4) clinical application of the hybrid abutment and the temporary PMMA crown; (5) intraoral scan of the hybrid abutment; (6) CAD of the final crown with automated margin line design using AI; (7) milling, sintering and characterisation of the final MZC; and (8) clinical application of the MZC. The outcome variables were mathematical (quality of the fabrication of the individual zirconia abutment) and clinical, such as (1) quality of the marginal adaptation, (2) of interproximal contact points and (3) of occlusal contacts, (4) chromatic integration, (5) survival and (6) success of MZCs. A careful statistical analysis was performed.

**Results:**

90 patients (35 males, 55 females; mean age 53.3 ± 13.7 years) restored with 106 implant-supported MZCs were included in the study. The follow-up varied from 6 months to 3 years. The quality of the fabrication of individual hybrid abutments revealed a mean deviation of 44 μm (± 6.3) between the original CAD design of the zirconia abutment, and the mesh of the zirconia abutment captured intraorally at the end of the provisionalization. At the delivery of the MZCs, the marginal adaptation, quality of interproximal and occlusal contacts, and aesthetic integration were excellent. The three-year cumulative survival and success of the MZCs were 99.0% and 91.3%, respectively.

**Conclusions:**

AI seems to represent a reliable tool for the restoration of single implants with MZCs cemented on customised hybrid abutments via a full digital workflow. Further studies are needed to confirm these positive results.

## Background

In implant-supported digital fixed prosthesis, one ideal option today is the use of customised abutments [1–3]. These custom abutments, designed with computer-aided design (CAD) software and subsequently milled and sintered in zirconia, are cemented extraorally on titanium bonding bases. Once applied, they allow obtaining an ideal emergence profile, high compatibility with soft tissues and high aesthetics [4]. Above these customised abutments, it is possible to cement monolithic restorations [4, 5]. However, while several clinical studies show that the use of these abutments can represent an ideal solution for the fixed rehabilitation of the implant patient, not only in the anterior [4] but also in the posterior areas [6], some practical problems are related to this approach.

In modern digital protocols, the dentist must capture an intraoral scan of the implant scanbody as accurately as possible [6, 7], and the technician must carefully perform the replacement of the mesh captured by scanning with the implant library files on which to model [8]; furthermore, implant libraries within the CAD software must not present errors. However, working well in these phases may not be sufficient: in fact, during the extraoral cementation of the customised zirconia abutment on the bonding base, tolerances between the components may cause cementing errors [8, 9]. These errors, even if only a few degrees, can generate positional problems during the delivery of the customised abutment and the temporary restoration to the patient: these components will not be in the exact CAD-planned position in the mouth [9]. During the delivery of the temporary restoration, small adjustments in resin or polymethylmethacrylate (PMMA) can be tolerated at the level of interproximal contacts or occlusion, but these adjustments are not acceptable for monolithic ceramic restorations, such as zirconia [9, 10]. The definitive monolithic zirconia restorations cannot be retouched in the mouth [10, 11], so they must not show positional errors at delivery.

To overcome this problem, experienced dental technicians generally position the individual abutments already assembled on a three-dimensional (3D) printed model, with the implant analogues inserted, and scan them with a desktop scanner. This allows obtaining the relative position and anatomy of the abutments, including the margin line [12]. Although this is possible, it is an extra step that forces the technician to print a model, with related costs and problems [13], but above all to model the definitive zirconia restorations on meshes that are, by definition, surface reconstructions and geometric approximations of the scanned objects [9, 14].

These additional steps can now be avoided by using artificial intelligence (AI). AI is a wide-ranging branch of computer science concerned with building smart software or machines capable of performing tasks that typically require human intelligence [15, 16]. It is commonly defined as “the ability of a system to interpret external data, learn from them, and use those learnings to achieve objectives and goals through flexible adaptation” [15, 16]. Machine learning, as a subset and foundation of AI, is the ability of computer systems to perform specific tasks to approximate human cognition, without using explicit instructions, solely relying on patterns and mathematical models [17]. AI can represent a valuable addition to fixed implant prosthodontics. CAD software can be ‘instructed’ to save the stereolithographic (.STL) file of the individual abutment, modelled by the technician, in a specific folder, and then to retrieve it automatically when needed [9]. At the end of the provisional period, the dentist can capture a new intraoral impression of the hybrid abutment in the correct position, after removal of the temporary restoration, without worrying about the visibility of the abutment margins, which are generally subgingival. The mesh captured with this intraoral impression is then imported into the CAD software. The portion relative to the individual abutment, captured in the mouth, is automatically recognised and eliminated because it is replaced with the original. STL file of the zirconia abutment, previously modelled by the dental technician [9].

The advantage of this approach is two-fold. The technician can model on a library file much more accurately than on a mesh, which is always a geometric approximation [9, 14]. Additionally, the software can automatically detect the margin line, even if subgingival, and draw it without error, using AI [9, 15]. This allows the technician to model without regarding the margins, focusing only on tooth shape, volumes, and interproximal and occlusal contacts. This innovative approach, which exploits the AI of the software, allows the technician to save time, while reducing errors and costs of prosthetic therapy; it is not necessary to print the 3D model with the digital analogues, nor to scan it [13]. Moreover, this approach could extend the use of individual hybrid abutments even in the posterior sites. An additional advantage of this method is that it allows quantitative verification and measurement of the overall mathematical quality of the digital workflow, for the fabrication of individual zirconia abutments [9].

The purpose of this retrospective clinical study is to present a protocol for the use of AI to fabricate implant-supported monolithic zirconia crowns (MZCs) cemented on customised hybrid abutments, via a full digital workflow.

## Methods

### Patient selection and inclusion/exclusion criteria

The present retrospective study was based exclusively on data collected on patients who had been treated through prosthetic restoration of single locking-taper connection implants (Exacone®, Leone, Florence, Italy) with MZCs in the posterior jaws (premolars and molars), between June 2016 and April 2019, in a single dental centre. The MZCs were made through a full digital workflow, without therefore producing any physical model, and were supported in all cases by individual hybrid abutments, composed of an upper portion in zirconia, cemented on a titanium bonding base. The data collected in the electronic medical records and necessary for the inclusion of the patients in the present study consisted of intraoral scans captured from the patient during the different work phases, the CAD scenes generated at the end of modelling the different components (individual abutments, temporary crowns in PMMA and definitive crowns in zirconia), and clinical, radiographic and photographic data normally collected during prosthetic implant treatment. All patients had previously signed a generic informed consent to prosthetic implant treatment, and a condition for inclusion in this study, on the nature of which all patients were properly informed, was signing a further specific consent. Exclusion criteria from this retrospective study were patients treated with implants produced by different manufacturers, or with multiple implants supporting fixed restorations such as bridges and full-arch prostheses; all patients treated with analog methods (i.e., through the capture of analog impressions with trays and conventional materials) or not full digital techniques; patients who were treated with full digital techniques, but through the printing of 3D models and modelling of zirconia copings to be layered with ceramic; all patients who had no opposing dentition; and patients who did not give consent for enrolment. The study was conducted in full accordance with the principles of the Helsinki Declaration on Human Experimentation of 1975 (and Revision of 2008) and was approved by the Ethics Committee of Sechenov University in Moscow, Russia.

### Clinical and laboratory procedures

The procedures for prosthetic rehabilitation consisted of the following eight phases (four clinical and four laboratory), based on the proprietary protocol #ScanPlanMakeDone, as already described in a previous scientific work [9].

**Fig. 1:**
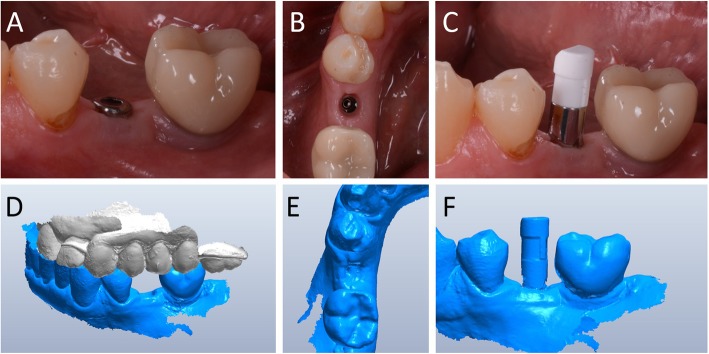
First intraoral scan of a second mandibular premolar. (**A**) The healing abutment in position. (**B**) The healing abutment is removed for the scan. (**C**) The scanbody is placed in position. (**D**) Intraoral scan of the master model without the scanbody and the antagonist arch. (**E**) The mucosal collar. (**F**) The implant scanbody in position

**Fig. 2:**
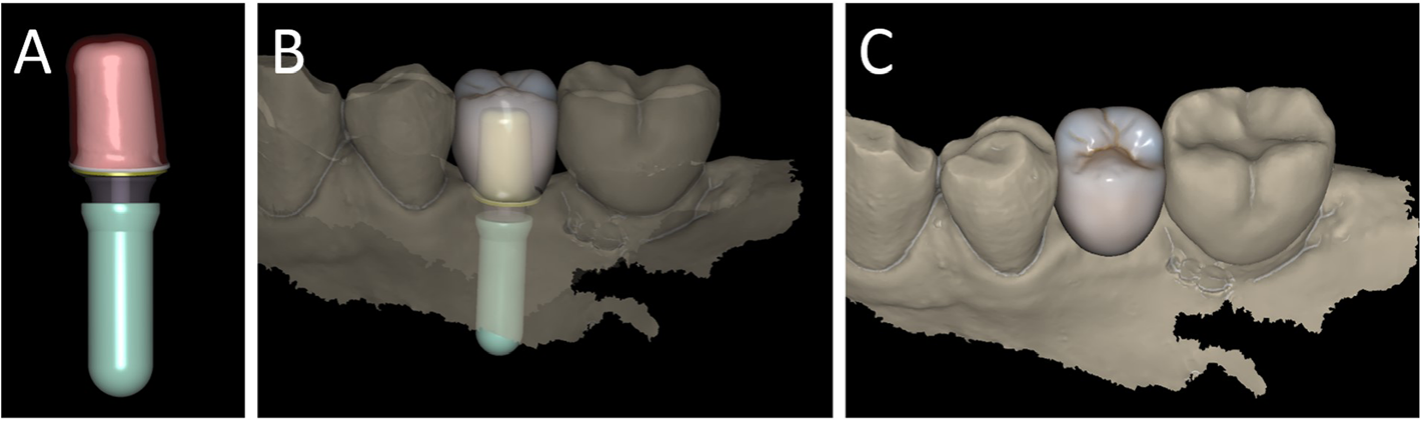
Computer-assisted-design (CAD) of the individual abutment and the provisional crown. (**A**) The individual abutment is modelled in CAD and saved in a dedicated folder of the software. (**B**) The individual abutment and the provisional crown. (**C**) Photorealistic rendering of the provisional crown

**Fig. 3:**
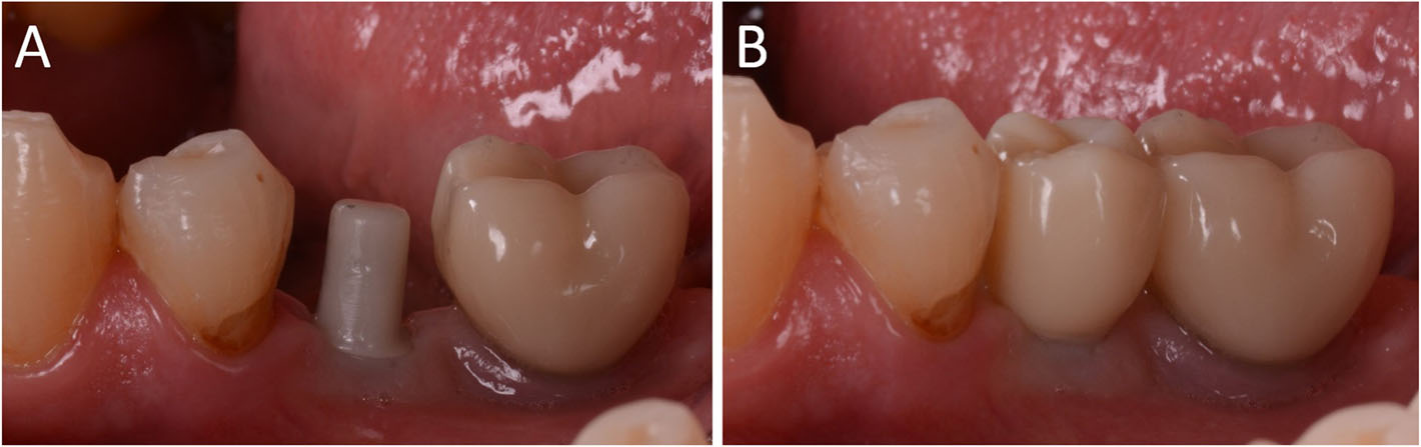
Delivery of the individual hybrid abutment and the provisional crown. (**A**) The individual hybrid abutment is placed. (**B**) The provisional polymethyl-methacrylate (PMMA) crown is cemented over it

**Fig. 4:**
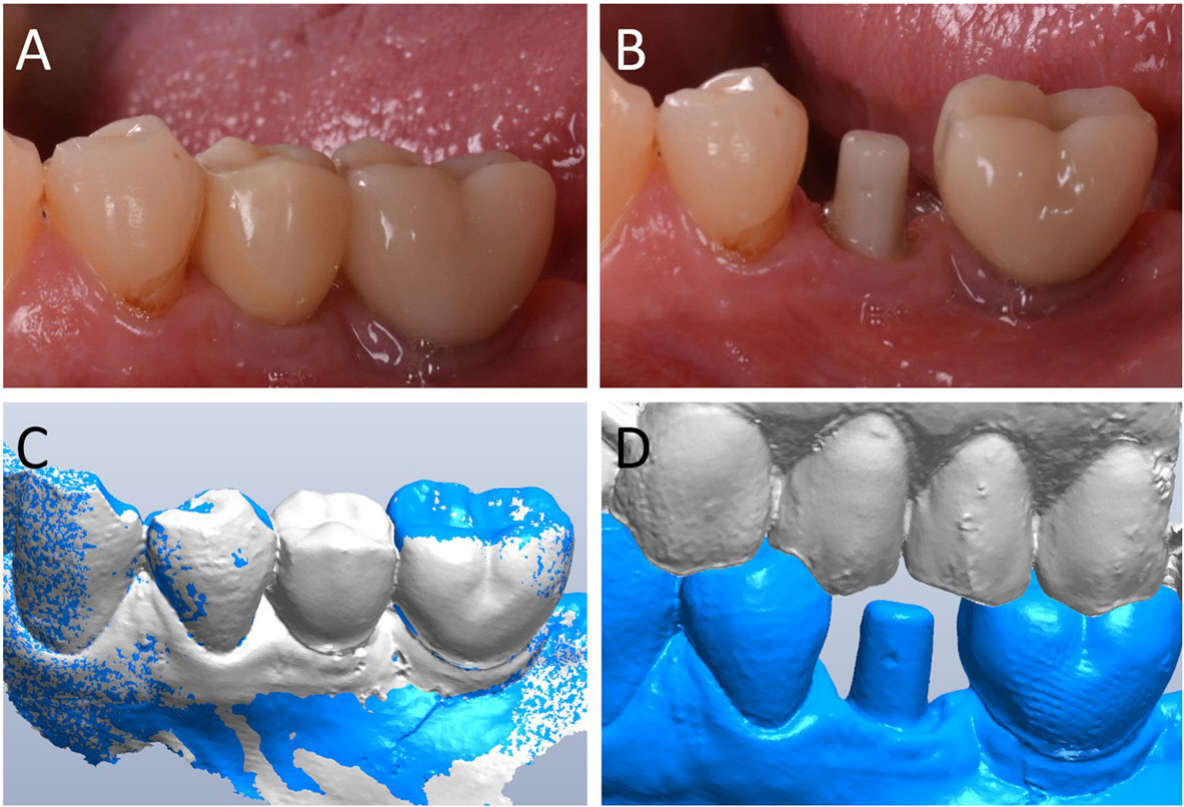
When the provisionalization period ends, a second digital impression is taken with and without the provisional crown, in order to capture the bite, the intraoral position of the zirconia individual abutment, and the status of the soft tissues. (**A**) The PMMA crown in position after a period of 2 months. (**B**) The individual hybrid abutment 2 months after placement. The abutment is carefully cleaned by any residual cement particles, the soft tissues are mature and everything is ready for the intraoral digital impression. (**C**) The first impression of the master model is captured with the functionalized provisional in position, in order to provide the dental technician information on the anatomy of the provisional and its occlusal limits. (**D**) after the removal of the provisional, the intraoral digital impression of the master model with the abutment in position is captured

**Fig. 5:**
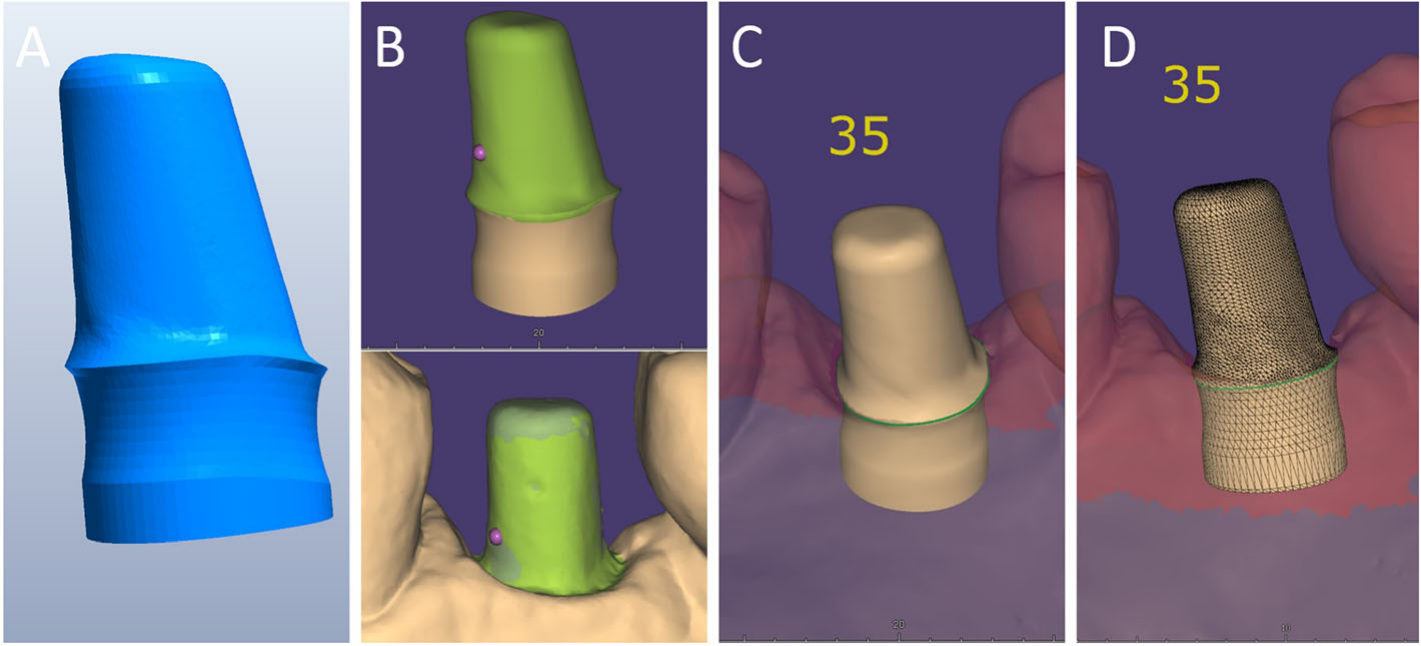
Artificial Intelligence (AI) application in fixed implant prosthodontics. (**A**) The original .STL file of the CAD design of the individual abutment, which was previously saved in a dedicated folder, is recalled by the system. (**B**) The original CAD design of the abutment is superimposed on the mesh captured intraorally. (**C**) Automatic margin line detection. (**D**) Details of the original CAD model, in which the abutment margins, although subgingival, are clearly represented and visible

**Fig. 6:**
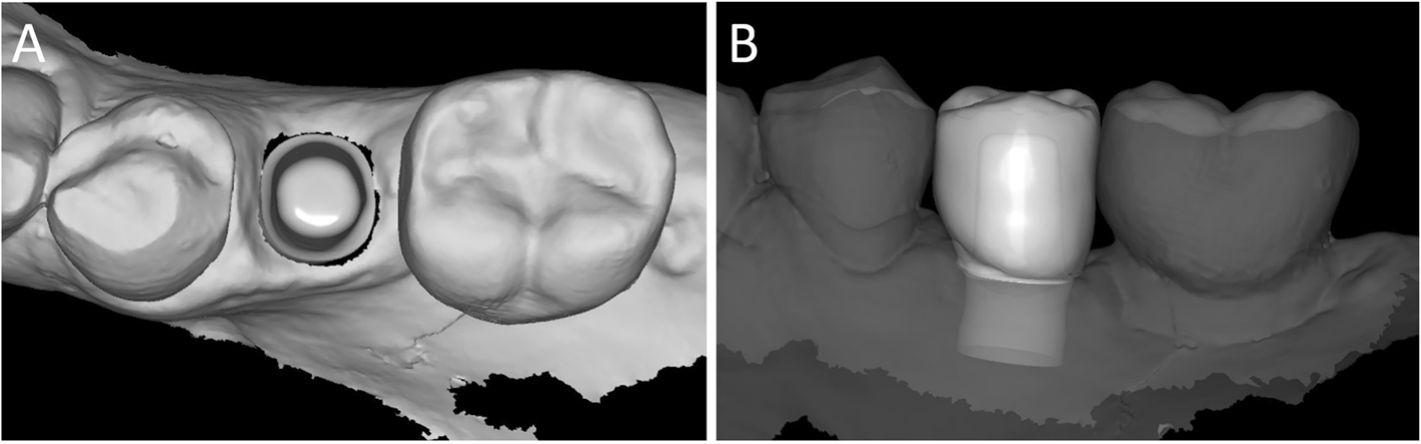
The final CAD scene. (**A**) details of the individual abutment in position: the technician will model the final zirconia crown over it; the dental technician can model the final crown on a library file. (**B**) CAD design of the final crown. The software is capable to automatically detect the margin line, because the final crown is modelled on the original CAD design of the individual hybrid abutment in the correct position, and not over a mesh. The technician can, anyway, modify the margin line, but this is not recommended because the software is able to detect it perfectly, better than the human eye

**Fig. 7:**
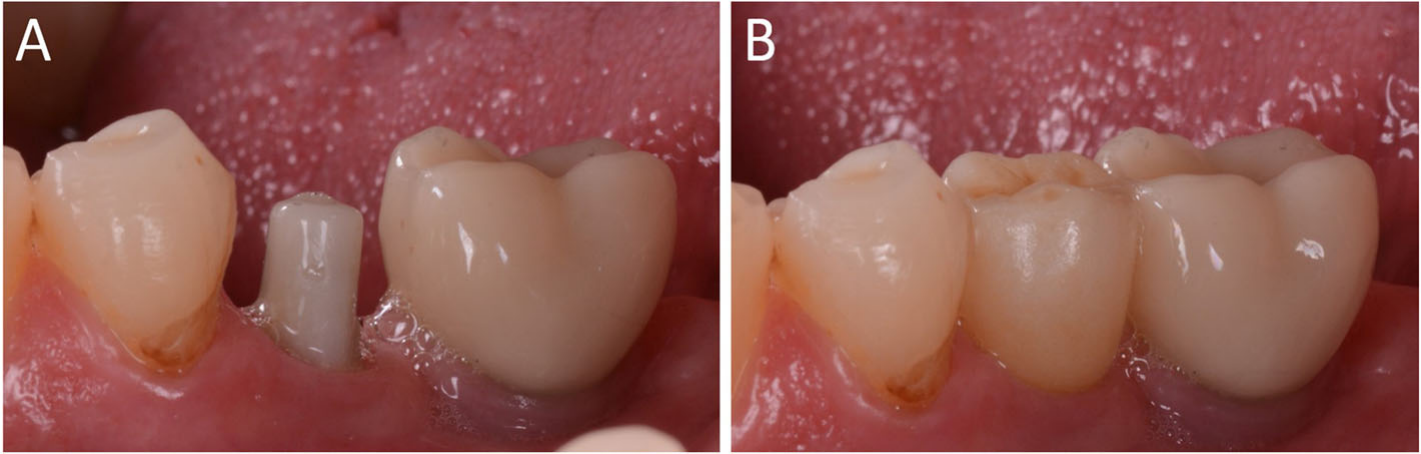
Delivery of the final zirconia crown. (**A**) Details of the individual hybrid abutment at the delivery of the final crown. (**B**) The final zirconia crown and its aesthetic integration

1. Capture of the first optical impression with the CS 3600® intraoral scanner (Carestream Dental, Atlanta, GA, USA) using the ‘implant’ module (Fig. 1). Once the healing abutment was removed, the dentist took an impression of the master model without a scanbody, to capture the mucosal collar and, where present, the adjacent teeth. The first phase of the capture of the impression was completed with acquisition of the antagonist model and the bite. Then, the dentist selected the area of ​​the mucosal collar on the master model and, after positioning the implant scanbody of the same diameter of the implant, captured it. Care was taken to capture the whole scanbody. The scanner perfected the image, and after a careful verification that confirmed the quality of the impression, the clinician removed the implant scanbody, repositioning the healing abutment. The. STL files derived from the optical impression were sent to the dental laboratory.
2. Modelling of the individual abutment and temporary crown in CAD software (Valletta®, Exocad, Darmstadt, Germany) (Fig. 2). The technician imported the. STL files into the CAD software and, after placing the order, replaced the mesh of the scanbody captured by the dentist with the corresponding library file, through superimposition by points and surfaces, using the software’s powerful algorithm. Having carried out this replacement, the technician selected the bonding base from a range of possible options (4-mm height straight base, or 6-mm height straight or angled base) according to the specific clinical indications of the case. Next, the technician modelled the individual abutment in its lower and upper portions, and the temporary crown to be cemented over it. The individual abutment was integral and did not show any screw holes, as the implants used in this study had a screwless, Morse-taper implant–abutment connection. However, the technician modelled a small hole at the top of the abutment to facilitate the outflow of cement during the cementation. The abutment and crown files were saved in. STL format and were ready for production. The. STL file of the individual abutment was saved in a specific folder created within the CAD software, containing all the modelling of individual abutments of all treated patients. Each model carried the patient’s name in addition to the number corresponding to the dental element that was prosthetically rehabilitated.
3. Production of the individual abutment and temporary crown. The individual abutment was milled in zirconia with a powerful five-axis milling machine (DWX-51®, DGShape a Roland Company, Hamamatsu, Japan) and then sintered in an oven (Tabeo®, Mimh-Vogt, Stutensee, Germany). At this point, the abutment was cemented by the dental technician on the titanium bonding base chosen during CAD modelling, which was purchased by the implant manufacturer. The cementation of the two components of the future individual hybrid zirconia–titanium abutment (upper portion individualised in zirconia on a standard titanium bonding base) occurred in the laboratory, under 4.5x magnification (Zeiss 4.5x®, Zeiss, Oberkochen, Germany), using a resin cement (Bifix SE®, Voco, Cuxhaven, Germany), taking care not to make macroscopic errors, due to the inevitable presence of minimal tolerances between the parts. The temporary crown was milled in PMMA from the modelling. STL file, using the same milling machine. It was then characterised, polished and fitted onto the individual abutment for control of marginal closure. At the end of these procedures, the dental technician sent the individual hybrid abutment and temporary crown to the dentist for clinical application.
4. Clinical application of the individual hybrid abutment and the temporary PMMA crown (Fig. 3). After removing the healing abutment, the dentist placed the hybrid abutment in the correct position, using the temporary crown as a guide. The abutment was engaged in the positional hexagon and activated with a percussion hammer, as previously described for locking-taper implants [9]. The margins were generally positioned 1 mm subgingival or juxtagingival, and the lower portion of the abutment generated minimal compression on the soft tissues, with ischaemisation of the peri-implant mucosa. After the abutment activation, the dentist verified the quality of the modelling, with particular regard to interproximal and occlusal contacts. In all cases in which small adaptations were necessary, they were performed directly in the mouth, until the final polishing of the provisional crown and its cementation on the individual abutment with a temporary cement (Tempbond®, Kerr, Orange, CA, USA). The provisional crown lasted from one to 2 months, to verify the adaptation of the implant under load and the maturation of the mucosal tissues. At the end of this period, the patient was recalled for the final scan.
5. Scanning the position of the hybrid abutment in the mouth (Fig. 4). The patient was recalled and subjected to a new intraoral scan of the dental arches with the CS 3600® scanner. Scanning was performed in ‘restoration’ mode. The first scan of the master model was captured with the temporary restoration in situ, to provide the dental technician with additional information on the anatomical limits of the modelling. The scan was completed with the capture of the opposing arch and the bite. Then, the dentist removed the temporary crown, and captured a fourth scan of the zirconia abutment in position, with the tissues conditioned by the temporary restoration. In this scan, the dentist focused on capturing the entire zirconia prosthetic abutment, and on capturing the contact points with adjacent teeth, where present. The. STL files or meshes derived from this scan were sent to the laboratory, and the temporary crown was cemented again on the abutment.
6. In the laboratory, the scan files were opened by the dental technician in the CAD software. First, the technician highlighted the area of the individual abutment on the mesh captured with the intraoral scan. Then the software was able to recall, within the specific folder containing all the modelling of the individual abutments of all the patients treated, the modelling corresponding to the patient in question, and to the implant in question. The original CAD file of the upper portion of the customized abutment was “extruded” like a model die, and “cut” or sectioned on the basis, in order to look like an “open” file, mimicking a mesh: in fact, Exocad® does not allow the dental technician to model on “closed” files (i.e., ready to be milled or printed). All the following procedures were performed using the intrinsic AI and the algorithms of the software. The software then replaced only the highlighted portion of mesh captured intraorally, with the corresponding. STL file of the original abutment modelling. The dental technician could test the quality of the overlap in micrometres by generating a colorimetric map. The original modelling file of the individual abutment was now integrated with the mesh of the master model, in the correct spatial position. Using intrinsic AI, the software was able to automatically trace the margin line of the implant abutment, though subgingival (Fig. 5). The technician could intervene on the design of the margin line, being able to modify it at will, but this was never necessary since the margin line drawn by AI was perfect in all cases. It was drawn on a file modelled in CAD, and therefore extremely clear. The software was able to detect it automatically and to design it without any problems. At this point, the dental technician could shape the final crown (Fig. 6). By opting for a monolithic zirconia restoration, it was not necessary to draw any model to print in 3D. The technician had to focus solely on modelling the shapes and volumes of the tooth, interproximal contact points and occlusion. The final 3D modelling was saved in. STL format and ready for production.
7. Production of the definitive monolithic crown in translucent zirconia. The final modelling file was processed by the aforementioned five-axis milling machine (DWX-51®, DGShape a Roland Company, Hamamatsu, Japan) to obtain the MZC, which was infiltrated when still green for better characterisation, and subsequently sintered. The crown was polished and ready for clinical application.
8. Clinical application of the MZC (Fig. 7). The patient was called back to the dental clinic, and the temporary PMMA crown was removed and replaced with the definitive MZC. At the time of application, the dentist carefully checked the congruity of the shape and volumes of the tooth, the quality of the interproximal contact points and the occlusal contacts, and the quality of the marginal fit and closure. Control of the marginal fit and closure of the restoration took place both clinically and radiographically. Before cementation with temporary cement (Tempbond®, Kerr, Orange, CA, USA), the MZC was positioned and an endoral x-ray was taken. Clinical control of the marginal closure occurred under 4.5x magnification (Zeiss 4.5x®, Zeiss, Oberkochen, Germany), with physical probing of the circumference of the crown with a periodontal probe to intercept any misfits, gaps or undercuts. Occlusal control was very careful and performed with occlusion papers so that any light precontacts were polished. At the end of these checks, and once the aesthetic adaptation of the restoration and the colour had been verified, the crown was cemented and the patient was included in a program of annual checks (two professional hygiene sessions a year, with one planned every 6 months).

### Study outcome variables

The variables investigated in this study were of two types: mathematical and clinical. First, the mathematical quality of the protocol for the fabrication of the individual hybrid abutments was inspected, by means of the superimposition of the original CAD design of the upper portion of the abutment over the mesh of the actual zirconia abutment captured intraorally with digital impressions. This type of mathematical evaluation made it possible to calculate the mean spatial error in the production (milling/ sintering) of the individual zirconia abutment, and to highlight the areas were any dimensional errors were concentrated. Then, the clinical variables (divided into primary and secondary clinical variables) were investigated. The clinical outcome variables were divided into primary and secondary not by importance, but because they were evaluated at different times.

### Mathematical quality

The mathematical quality of the protocol for the fabrication of the individual hybrid abutments was controlled when the superimposition of the original CAD design of the upper portion of the abutment over the mesh of the actual zirconia abutment captured intraorally was performed. In fact, the CAD files of the drawing of the individual hybrid abutment, and the mesh of the actual position of the zirconia abutment captured intraorally were saved as individual STL files after the superimposition in Exocad®, and were imported in a powerful reverse-engineering (Studio2012®, Geomagics, Morrisville, NC, USA). This software was employed to calculate the distance (mean ± SD in μm) between the visible, supramucosal surfaces of these two different STL files. In order to avoid any error given by the presence of the soft tissues, the calculation was limited to the area above the soft tissues. This type of mathematical evaluation made it possible to calculate the spatial error in the production (milling/ sintering) of the individual zirconia abutment. Finally, a digital colorimetric map was generated by the software, in order to better highlight the spatial deviations between the different files, at different levels. The threshold was set at 30 μm, so that any deviation < 30 μm was represented in green colour; deviations > 30 μm were represented in blue colour (dark blue for major deviations).

### Clinical quality

1. Primary clinical variables (evaluated immediately on delivery of the MZC and related to the quality of the prosthetic implant restoration).

2. Secondary clinical variables (evaluated from 6 months to 3 years later and related to the survival and success of the MZC over time).

These variables were investigated by a prosthodontist and a periodontist, both experts.

### The primary clinical variables were

#### 1A. Quality of the marginal adaptation and closure

The quality of the marginal closure was clinically investigated through visual inspection with magnifying glasses (Zeiss 4.5x®, Zeiss, Oberkochen, Germany) and tactile analysis through a circumferential probing at the crown positioned on the individual abutment, with a periodontal probe, and finally, through radiographic analysis with evaluation of endoral RX. The purpose of this analysis was to assess the presence of any defects, misfits, gaps or undercuts.

#### 1B. Quality of interproximal contact points

Quality control of interproximal contact points with adjacent teeth, where present, was performed visually and using dental floss.

#### 1C. Quality of occlusal contacts

Occlusion control was performed clinically, using articulating papers (Bausch Articulating Paper®, Bausch Inc., Nashua, NH, USA).

#### 1D. Chromatic and aesthetic integration

The quality of the colour integration of the restoration was assessed visually.

At the end of the evaluation, the two operators who evaluated the quality of the MZCs assigned each restoration a score from 1 to 5 (with 5 as the highest value, expressing fully satisfactory quality; 4 for satisfactory quality; 3 for acceptable quality; and 2 and 1 as the lowest values, expressing a restoration of unsatisfactory quality) for each of the aforementioned parameters. If even one of the four parameters investigated by the two operators was of unsatisfactory quality, and had therefore received a grade < 3, the definitive restoration was not cemented and was sent back to the dental technician for remaking. This happened if the marginal adaptation of the restoration was unsatisfactory, in the presence of gaps or over-boundaries; if the interproximal contact points were missing or unsatisfactory, to avoid stagnation of food and hygiene problems; where there were excessive occlusal contacts that could not be eliminated by simple polishing, or a lack of appropriate occlusal contacts (infraocclusion restoration); and where the colour of the MZC did not fit the context of the patient’s oral cavity, and the aesthetic integration was then unsatisfactory.

### The secondary clinical variables were

#### 2A. *Survival of the restoration*

An implant-supported restoration was called a ‘survivor’ when functioning properly until the final check-up [18]. It was defined as ‘failed’ if it went into failure (for example, failure of the implant, or fracture of the monolithic crown in translucent zirconia) during the whole post-delivery period [18].

#### 2B. *Success of the restoration*

An implant-supported restoration was defined as ‘successful’ if it did not present any complication during the whole period after delivery [9, 19]. The restoration was considered ‘unsuccessful’ if, although not failed and still physically present in the mouth, it presented or had presented during the period of follow-up any biological complications (peri-implant mucositis with gingival swelling, discomfort, and/or bleeding [20]; and/or peri-implantitis with pain, suppuration, bleeding, and/or marginal bone resorption [21]); prosthetic (mechanical) complications [22, 23] (problems affecting pre-formed components sold by the manufacturer, such as the loss of connection between abutment and implant, or fracture of the fixture or bonding base); or technical complications [23, 24], (problems affecting the components designed by the dental technician, such as debonding of the upper portion of the zirconia abutment from the titanium base, fracture of the upper portion of the zirconia abutment, or decementation or chipping of the MZC [2, 10, 11]).

These variables were investigated during the twice-annual scheduled check-ups, during which the patients underwent professional oral hygiene sessions, by the two aforementioned clinicians (prosthodontist and periodontist).

### Statistical analysis

The data related to the present study were collected in the patients’ electronic medical records and used for statistical analysis. The statistical analysis was first descriptive, based on the demographic characteristics of the patients (age, gender, smoking habit and presence of bruxism) and the features of the restorations (location and position of the crowns, as well as type of bonding base used and implant diameter). Mean, standard deviation (SD), median, range and 95% confidence interval (CI) were calculated for quantitative variables, such as patient age and mathematical quality of the protocol for the fabrication of the individual hybrid abutments; absolute and relative (%) distributions were calculated for qualitative variables such as gender, smoking habit and parafunction, as well as location and position of crowns, type of bonding base and implant diameter. The Chi-square test was used to assess homogeneity or inhomogeneity within the groups, with a level of significance set at 0.05. With regard to the primary clinical outcomes of the study, i.e. the variables investigated at the delivery of the final crowns (marginal adaptation and closure, quality of the interproximal contacts, quality of the occlusal contacts, aesthetic outcome), the mean scores (±SD) given by the different independent observers (prosthodontist and periodontist) were calculated, as well as the incidence of complications or issues found within the different groups, with absolute and relative distributions. For the secondary clinical outcomes, i.e. the variables investigated during the scheduled twice-annual check-ups, the incidence of failures and complications were respectively calculated, and the cumulative survival and success of the implant-supported restorations were calculated using the life-table analysis of Cutler and Ederer [25]. Survival and success of the crowns were calculated at the restoration level; in the context of the calculation of success, even a single complication was sufficient to allocate the restoration into the group of failures.

## Results

### Patient population and implant-supported crowns

Ninety patients (35 males and 55 females; aged between 22 and 79 years, with a mean age of 53.3 ± 13.7 years, median 54 years, CI 95% 50.5–56.1 years) who had been restored with 106 implant-supported MZCs were included in the study. Among the 90 patients included, 15 were smokers and 22 were bruxists. The restorations were positioned in both arches (66 maxilla, 40 mandible), in the posterior sectors of the mouth (35 premolars, 71 molars). All implants featured a locking-taper implant–abutment connection, presenting a screwless self-locking connection between the abutment and fixture, with angle 1.5°. The restored implants were of different diameters (3.3 mm: five fixtures; 4.1 mm: 45 fixtures; 4.8 mm: 56 fixtures). All 106 restorations were supported by individual hybrid abutments; the chosen bonding bases in titanium were 4-mm straight (23 Tibase®, Leone, Florence, Italy), 6-mm straight (43 Multitech® straight, Leone, Florence, Italy) and 6-mm angled 15° (40 Multitech® angled, Leone, Florence, Italy). The distributions of the patients and restorations are summarised in Table 1 and Table 2. The distribution of the patients was homogeneous by gender (*p* = 0.133), but not homogeneous in terms of age (*p* = 0.0003), smoking habit (*p* <  0.0001) or bruxism (p = 0.0003): most were aged > 35 years, non-smokers and without parafunction. The distribution of the crowns was homogeneous by location (*p* = 0.071) and bonding base (*p* = 0.162), but not homogeneous in terms of position (*p* = 0.012) or supporting implant diameter (*p* <  0.00001): most were molars, and only a few 3.3-mm diameter implants were used.

**Table 1:** Distribution of the patients by gender, age at enrollment, presence of smoking habit and bruxism

	N°	p*
Gender		
*Males*	35 (38.9%)	0.133
*Females*	55 (61.1%)	
Age		
*< 35 years*	8 (8.9%)	0.0003
*35–55 years*	42 (46.7%)	
*> 55 years*	40 (44.4%)	
Smoke		
*Yes*	15 (16.7%)	< 0.0001
*No*	75 (83.3%)	
Bruxism		
*Yes*	22 (24.4%)	0.0003
*No*	68 (75.6%)	
Overall	90	–

^*^Chi-square test

**Table 2:** Distribution of the restorations by location, position, titanium bonding base, implant diameter and length

	N°	p*
Location		
*Maxilla*	66 (62.3%)	0.071
*Mandible*	40 (37.7%)	
Position		
*Premolar*	35 (33%)	0.012
*Molar*	71 (77%)	
Bonding base		
*Tibase®*	23 (21.7%)	0.162
*Multitech® straight*	43 (40.6%)	
*Multitech® angled 15°*	40 (37.7%)	
Diameter		
*3.3 mm*	5 (4.7%)	< 0.00001
*4.1 mm*	45 (42.5%)	
*4.8 mm*	56 (52.8%)	
Overall	106	–

^*^Chi-square test

### Mathematical quality of the protocol

**Fig. 8:**
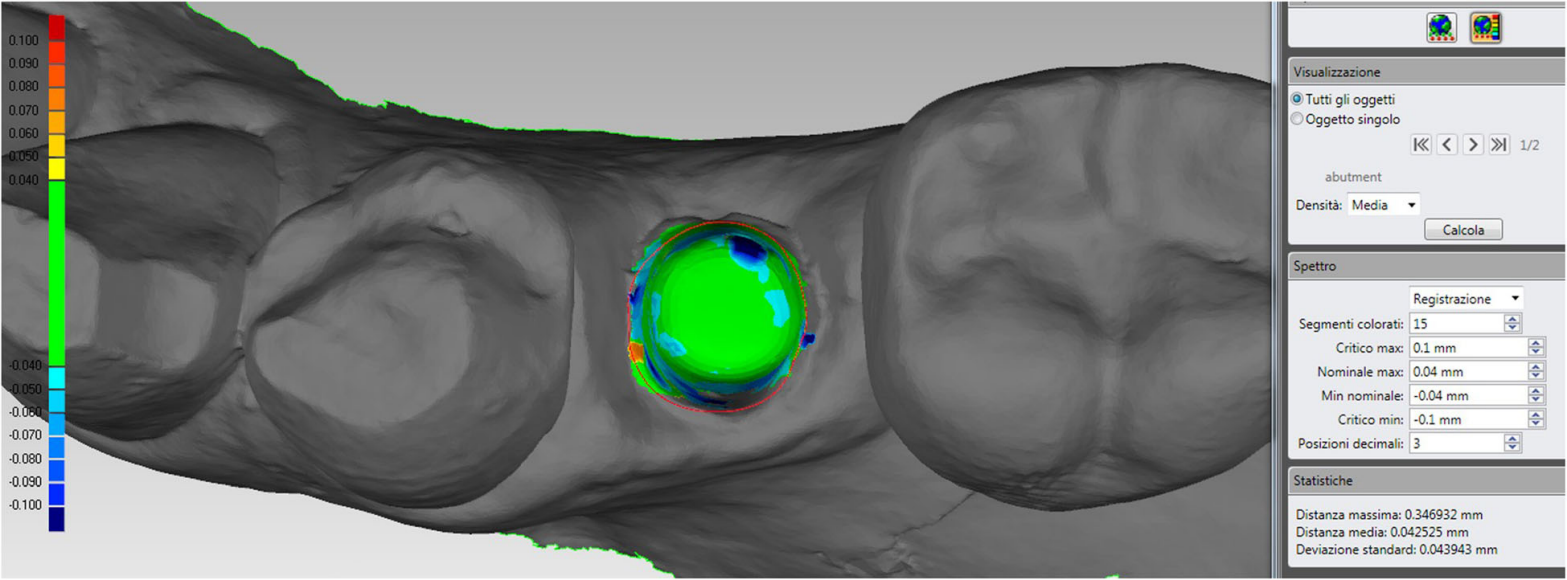
For the present case, the quality in the fabrication of the individual hybrid abutment was good, with a mean deviation of 42 μm (±43) between the original CAD design and the mesh of the zirconia abutment captured intraorally. It must be noted that the presence of a little hole on the top of the actual abutment (very useful to facilitate the outflow of the cement in excess, during the extraoral cementation in the laboratory of the upper zirconia portion on the titanium base), depicted here in dark blue, may increase the mathematical error; however, this is clinically not relevant. The software used for this calculations was a powerful reverse engineering (Studio 2012, Geomagics, Morrisville, NC, USA) able to detect deviations up to 1 μm

The evaluation of the quality of the protocol for the fabrication of individual hybrid abutments revealed a mean deviation of 44 μm (± 6.3; median 45; range 28–64; confidence interval 95% 42.9–45.1) between the original CAD design of the zirconia abutment, and the mesh of the zirconia abutment captured intraorally at the end of the provisionalization (Fig. 8).

### Primary clinical outcomes

The mean (±SD) scores given by the two independent operators, related to the four primary outcomes (quality of the marginal closure and adaptation, quality of the interproximal contacts, quality of the occlusal contacts and aesthetic integration) are summarised in Table 3. The issues encountered at the delivery of the final crowns are summarised in Table 4.

**Table 3:** Means (±SD) for the primary outcomes (quality of the marginal adaptation/ closure, interproximal contact points, occlusal contacts and aesthetic integration) of the study, as assigned by two experienced operators, using a score from 1 to 5 (with 5 as the highest value, expression of a fully satisfactory quality; 4 for a satisfactory quality; 3 for a quality acceptable; 2 and 1 as the lowest values, expression of a restoration of unsatisfactory quality)

	Prosthodontist Mean score (±SD)	Periodontist Mean score (±SD)	Overall Mean score (±SD)
Quality of the marginal adaptation/ closure	4.41 (0.7)	4.41 (0.7)	4.41 (0.7)
Quality of interproximal contact points	4.49 (0.7)	4.43 (0.6)	4.46 (0.6)
Quality of occlusal contacts	3.84 (0.8)	3.94 (0.9)	3.89 (0.8)
Chromatic and aesthetic integration	4.25 (0.7)	4.04 (0.6)	4.15 (0.7)
Overall mean score (±SD)	4.25 (0.8)	4.20 (0.7)	4.23 (0.7)

**Table 4:** Problems encountered at the delivery of the final monolithic translucent zirconia crowns, and rate of complications

Type of issue	Incidence	Complication rate
Marginal adaptation	2/106	1.8%
Interproximal adaptation	2/106	1.8%
Occlusal adaptation	6/106	5.6%
Chromatic integration	3/106	2.8%
Overall	10/106	9.4%

### Quality of the marginal closure

The marginal closure and adaptation of the final MZCs were checked clinically with visual inspection (under 4.5x magnification) and probing, as well as radiographically. The mean score (±SD) was the same for both the prosthodontist and the periodontologist, amounting to 4.41 (0.7). In almost all cases (102/106 crowns, 96.2%), the marginal adaptation was excellent, and the operators detected insufficient quality (score < 3) in only four cases. In these cases, the crowns were sent back to the technician for remodelling and milling. The incidence of complications therefore amounted to 1.8%.

### Quality of interproximal contacts

The quality of the interproximal contacts was excellent in almost all cases, with a mean score (±SD) of 4.49 (0.7) and 4.43 (0.6) for the prosthodontist and the periodontologist, respectively. However, in two crowns (1.8%), the contact points were rather weak and loose, with a score < 3. To avoid food impaction, these crowns were sent back to the technician for remodelling and milling.

### Quality of occlusal contacts

The quality of the occlusal contact points was rather good, with a mean score (±SD) of 3.84 (0.8) and 3.94 (0.9) for the prosthodontist and the periodontologist, respectively. However, in six cases of the 106 (5.6%), the occlusal adaptation of the final crowns was not acceptable because of the presence of precontacts (five cases) or the absence of occlusal contacts (infraocclusion, one case). In the cases of precontacts, two crowns were retouched by polishing the cusps before cementation, and were applied without issues; in three crowns, however, the occlusal precontacts were marked such that it was not possible to polish and apply them. These crowns were therefore sent back to the technician for remodelling and milling, exactly as in the case of the crown characterised by the absence of occlusal contact, where remaking was needed.

### Aesthetic integration

From the chromatic perspective, the results were excellent, with a score of 4.25 (0.7) and 4.04 (0.6) for the prosthodontist and the periodontist, respectively. Overall, the chromatic and aesthetic integration of the monolithic translucent zirconia crowns was good, with only three restorations (2.8%) scoring < 3. In these cases, the crowns were not remade, but sent back to the technician for better characterisation.

### Secondary clinical outcomes

The secondary outcomes of the study, i.e. the survival and success of the restoration, were evaluated during the scheduled twice-yearly follow-up sessions, by the same operators involved in the evaluation of the primary outcomes.

### Survival of the restorations

**Fig. 9:**
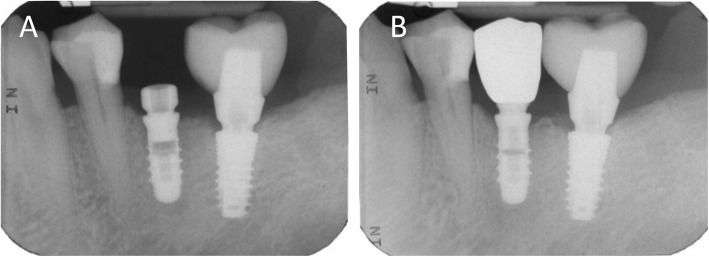
Radiographic controls before and after the prosthetic restoration. (**A**) Endoral periapical radiograph taken at the beginning of the restorative process, before the first intraoral scan, with the healing abutment in position. (**B**) Three years later, the crown is in function and seated with high precision over the abutment, with little or no gap

During the first year after the delivery of the final restorations, only one implant-supported MZC (a maxillary molar) was lost. This failure occurred 2 months after the delivery of the final crown, and was caused by the loss of the supporting implant, which was extra short (6.5 mm) and inserted in a smoker and bruxist. The supporting implant showed marked and progressive bone loss, in the absence of any clinical signs of infection. No further implant failures were observed, and no fractures of the MZCs occurred. The cumulative survival rate of the implant-supported restorations 3 years after the delivery of the final MZCs was 99.0% (Fig. 9), as reported in the life-table analysis of Cutler and Ederer (Table 5).

**Table 5:** Cumulative survival rate of the implant-supported MZCs by means of the life-table analysis of Cutler and Ederer. An implant-supported MZC was defined “survivor” if still in function, at the end of the different time intervals

Time interval (months)	Implant-supported crowns at the start of the interval	Drop-outs during the interval	Implant-supported crowns at risk	Failures during the interval	Survival rate within the period (%)	Cumulative survival rate (%)
0–6	106	2	104	1	99.03%	99.03%
6–12	94	1	93	0	100%	99.03%
12–18	84	0	84	0	100%	99.03%
18–24	75	1	74	0	100%	99.03%
24–30	69	0	69	0	100%	99.03%
30–36	40	0	40	0	100%	99.03%

### Success of the restorations

After the delivery of the final MZCs, among the 105 surviving restorations, only a few biologic and prosthetic complications were reported. Two biologic complications were reported, both peri-implant mucositis, for an overall incidence of 1.9%. Prosthetic complications were slightly more frequent, with six adverse events registered, for an incidence of 5.7%. Two prosthetic complications were mechanical in nature, with a loss of connection between the hybrid abutment and the fixture occurring in two crowns. These abutments were repositioned and reactivated, and no further issues were reported for these restorations; the complication was defined as minor in nature because it did not require any intervention from the technician. In two additional patients, unfortunately, the upper portions of the hybrid abutment (part in zirconia) decemented from the bonding bases. These complications were technical in nature and required a new extraoral cementation of the individual abutments on the titanium bases. These procedures were performed after careful cleaning and sandblasting of the bonding bases (with the aim to increase the adhesion) and required the intervention of the technician, so they were defined major in nature, also because the removal of the bonding bases from the fixtures was not easy for the dentist. Finally, two MZCs decemented from the hybrid abutments. In these cases, recementing them was sufficient. These last two complications were technical and minor in nature, as they did not require any intervention from the technician. The cumulative success rate of the implant-supported MZCs restorations 3 years after the delivery was 91.3%, as reported in the life-table analysis of Cutler and Ederer (Table 6).

**Table 6:** Cumulative success rate of the implant-supported MZCs by means of the life-table analysis of Cutler and Ederer. An implant-supported MZC was defined as “successful” in the absence of any biologic and/or prosthetic complication, at the end of the different time intervals

Time interval (months)	Implant-supported crowns at the start of the interval	Drop-outs during the interval	Implant-supported crowns at risk	Failures during the interval	Survival rate within the period (%)	Cumulative survival rate (%)
0–6	106	2	104	5	95.2%	95.2%
6–12	94	1	93	1	98.9%	94.1%
12–18	84	0	84	0	100%	94.1%
18–24	75	1	74	1	98.6%	92.7%
24–30	69	0	69	1	98.5%	91.3%
30–36	40	0	40	0	100%	91.3%

## Discussion

Less than a decade after breaking the Nazi encryption machine Enigma and helping the Allied Forces win World War II, mathematician Alan Turing changed history a second time, posing himself a simple question: ‘Can machines think?’ In 1950, with the paper titled ‘Computing Machinery and Intelligence’, Turing established the fundamental goal and vision of AI: to replicate or simulate human intelligence in machines [15, 26, 27]. Several years later, the first famous success of AI was that of Deep Blue, an IBM machine, that defeated the reigning chess champion Garry Kasparov. Although the first meetings were won by Kasparov, the continuous improvements brought to the learning system of Deep Blue allowed the machine to achieve victory in successive games. The victory, as declared by Kasparov, was possible because “the machine has reached such a high level of creativity, that goes beyond the knowledge of the player”. The foundation of AI is machine learning, a branch of computer science that builds algorithms to solve problems, guided by statistics and data [16, 17, 26, 27].

The concept of AI has evolved over time. From the fascinating but rather cinematographic idea of a “strong” AI in which super-intelligent robots (like the ones from *Westworld* or *Star Trek: The Next Generation*) overrun humanity, able to solve any problem, it has become something more “narrow” but concrete: basically, a way to construct algorithms that can learn from data and make predictions [26–28]. AI can today be defined as a branch of computer science that allows the programming and design of both hardware and software systems with certain characteristics that are typically considered human, such as visual, spatio-temporal and decision-making perceptions [26, 27]. “Narrow” or as it is sometimes called “weak” AI, therefore, is focused on performing single tasks extremely well, with several examples in daily life (from Google Search to Siri, Alexa and other personal assistants; and from IBM’s Watson to self-driving cars) [17, 27].

In the medical field, AI uses algorithms and software applications to approximate human cognition in the analysis of complex data, approaching levels of human expertise, changing the role of computer-assisted diagnosis from a ‘second-opinion’ tool to a more collaborative one [28]. The development of AI applications is already remarkable, particularly in radiology and 3D imaging, as an aid to human clinicians in diagnostic and treatment planning, and recently AI has been integrated into image processing software and CAD, with promising results [29].

AI systems can also be extremely useful in dentistry, as their common feature is that they need data to be processed to build algorithms useful for determining actions [17, 18, 28, 29], and the dentist produces a large amount of digital data that can be extremely useful to take advantage of AI benefits [17].

In the dental world, in fact, a real revolution is in progress, determined by the advent of digital technologies [30]. Intraoral [6, 7, 14], desktop [31] and face scanners [8], cone beam computed tomography [32] and digital condylographs allow acquiring a huge amount of 3D data useful for patient virtualisation [8]. This easily accessible data [17] can be used by computers for many purposes, not only to perform different CAD modelling (surgical [8, 33], prosthetic [2–5, 10, 30] and orthodontic [34]) that will then be produced physically for clinical use but also to instruct the same software so that it ‘learns’ certain mechanisms, and can therefore respond or act automatically, with an overall benefit for the workflow in terms of reducing processing times and costs [9].

In the field of implant prosthodontics, for example, the new digital workflows involve the use of intraoral scanners to capture the position of the implants through the scanbody, the digital version of the old implant transfer [6–9]. The accuracy of intraoral scanners is high today [6, 14], and these tools allow replacing the classic impression with trays and materials, with benefits for the patient and the entire prosthetic workflow [30]. Taking the impression is easier for the operator, involves less discomfort for the patient, and takes place in greater comfort and in a shorter time, with reproducible results [9, 14]. Communication with the laboratory is facilitated, and costs and time can be reduced. However, the introduction of digital technologies implies the need to adopt new protocols, and this is not always easy for the dentist and dental technician, especially when they are ‘native analog’.

The optical impression is transferred, generally in. STL format (sometimes in proprietary format) to the laboratory, which replaces the scanbody mesh with the corresponding library file, coupled to the whole set of files of the different bonding bases available [9, 30]. The dental technician can therefore choose to model an individual zirconia abutment, which will be milled, sintered and extraorally cemented onto the chosen titanium bonding base. This approach is probably the best from a clinical viewpoint, because the individual zirconia abutment makes it possible to model the emergence profile in an ideal way, is highly aesthetic and pleases the mucosal tissues, with excellent biological integration [2–6, 9–11, 30, 35].

However, the full digital workflow that involves the use of individual abutments, even in the presence of mathematically perfect libraries, presents pitfalls. In particular, a very delicate moment is the cementation of the upper zirconia portion of the hybrid abutment on the titanium bonding base [9]. This cementing takes place outside the mouth, in the laboratory, and although the technician pays the utmost attention to it, it is possible and even probable that the tolerances between the components determine position errors, i.e. a minimum degree of rotation between the components, at least compared to the original CAD project. This situation leads to incomplete correspondence between the CAD project and the intraoral situation at the time of the clinical application of the hybrid abutment [9]. This may not be a problem at the time of insertion of the temporary restoration in PMMA, which can be adapted through small adjustments and polishing, but certainly is a greater problem when working with definitive zirconia restorations, which by definition cannot be retouched in the mouth once applied [2–6, 9–11, 30].

In the present retrospective clinical study, which represents the development of a previously published work [9], we have presented a clinical protocol that is able to solve these problems in a simple and very predictable way, and with lower costs. This protocol is based on a second intraoral scan, at the end of the provisional period, with the mucosal tissues suitably conditioned by the temporary restoration. The patient is recalled, the provisional is removed and an optical impression of the individual hybrid abutment in situ is captured, regardless of the margins of the preparation, which are generally subgingival. This mesh, loaded into the CAD software, is automatically recognised by the software AI and replaced by the original modelling file, stored in a special folder. The technician can therefore model the final crown directly on the original modelling file of the abutment (and not on a mesh, which by definition is a geometrical approximation of the object), which is transported to the correct spatial position, the actual position of the hybrid abutment in the mouth. Furthermore, the software can automatically trace the margin line, which is recognised by the AI. The technician can thus model the final restoration on an abutment with clear margins, with the margin line already traced by the computer, although subgingival, and perform modelling on a library file (not on a mesh). This guarantees maximum clinical precision and the elimination of several risk factors (scanbody scanning errors, intrinsic library errors and cementation errors) in a context of simplification of procedures. In our present study, with 90 patients included, AI was a reliable tool for the restoration of 106 single locking-taper implants with MZCs cemented on customised hybrid abutments via a full digital workflow. In fact, the protocol for the fabrication of individual hybrid abutments revealed a high mathematical quality and reliability, with a mean deviation of 44 μm (± 6.3) between the original CAD design of the zirconia abutment, and the mesh of the zirconia abutment captured intraorally at the end of the provisionalization project. At the delivery of the final MZCs, the marginal adaptation, quality of the interproximal and occlusal contacts, and aesthetic integration were excellent, with satisfactory high scores. Moreover, the incidence of failures and complications was low, with three-year cumulative survival and success of 99.0 and 91.3%, respectively.

An alternative to our present AI protocol, run by the most experienced dental technicians, is today the 3D printing of physical models in which laboratory analogues are inserted, on which the individual abutments are screwed. The whole model plus the abutments are then scanned with a desktop scanner, and the technician models the final restorations on a mesh. Following this protocol, the technician transforms implant abutments into natural abutments, solving the issues related to the extraoral assembly or cementation of the individual zirconia portion on the titanium base. To do so, however, more steps are introduced, such as the need to print a model, with relative uncertainties [13, 36] and an increase in time and cost of the therapy. In fact, it is well known that 3D-printed models may present issues, if not printed with high-quality and accurate machines, and it is clear that the manual positioning of analogues inside them can cause positional errors (not only rotational but also in height) [36]. Furthermore, modelling on mesh is not ideal: it is certainly preferable to model on library files.

Our clinical protocol and AI may simplify the procedures, eliminating a series of steps, and it may therefore help to extend the use of individual hybrid abutments even in the posterior sites, for the replacement of premolars and molars. This can be important from a mechanical point of view, as it could help reduce prosthetic complications. In fact, the direct cementation of fixed superstructures (crowns, bridges) on titanium gluing bases is still not adequately documented in literature, and may lead to the onset of mechanical complications [2, 37]. The titanium gluing bases, in fact, are prefabricated and have standard height/ thickness, therefore may not withstand in the medium- and long term, the occlusal forces transmitted by monolithic structures characterized by larger dimensions [2, 37]. In addition, with the present AI protocol, as previously reported [9] it is possible to mathematically evaluate and therefore to exactly quantify the degree of error, in the fabrication (milling/ sintering) of the individual zirconia abutment. In fact, using a reverse engineering software, it is possible to calculate the distance between the surface of the original CAD design of the zirconia abutment, and the mesh of the zirconia abutment captured intraorally at the end of the provisionalization project. This can give relevant information on the quality of the production process, and it may allow to identify issues; however, it must be noted that digital impressions themselves entail a certain degree of error [6], which could, in part, jeopardize this quality control.

It should be stressed that the present AI protocol seems to work particularly well with the Morse taper connection implants used in this study. These implants do not have a connecting screw between the bonding base and fixture, but the engagement is a locking taper, with an angle of approximately 1.5 ° between the parts [23, 24]. This ‘cold fusion’ allows, in addition to the mechanical and stability advantages of the connection that are well described in the scientific literature [23, 24], modelling integral abutments without any hole for a passing screw, which does not exist. This strengthens the abutment, but also facilitates the task of the AI system. Most implant systems on the market today have instead a screw for the assembly of the bonding base and therefore of the individual abutment on the implant; the presence of the screw hole can weaken the implant abutment [38], causing difficulties in modelling where the screw hole is angled. Moreover, it makes the correct extraoral cementation of the zirconia portion on the titanium bonding base more difficult, and could potentially make the task of the AI system of the CAD software more challenging. Having ‘full’ integral abutments, as for locking-taper connection implants, facilitates the task of the software and paves the way for the AI system [9, 30]: these abutments are drawn with a very small hole in the head, designed only to facilitate the correct outflow of the cement during cementation. This hole is much smaller than a screw hole. However, the removal of hybrid conometric abutments can be difficult once activated, and this may represent a limitation. Another limitation is the absence of long-term clinical data on the reliability of customised zirconia abutments, particularly in the posterior areas of the jaws [39]. Moreover, the present protocol assumes that the hybrid abutments, once milled, sintered and assembled, are not modified in the laboratory: otherwise, the AI of the software may encounter difficulties in coupling the modelling file with the mesh captured in the mouth. In addition, the AI application presented here has to be considered “narrow” as the product of the application of specific algorithms and therefore “weak” in nature; this can be considered a further limitation of the present study. In fact, the only tasks performed automatically by the software are the recovery of the CAD file originally modeled by the dental technician, and the replacement of the mesh captured in the mouth with it. The automatic identification of the margins is itself to be considered a “narrow” application. It would be totally different if the software were able to automatically model the individual abutments, based on specific skills or knowledge acquired through deep learning: that would be an example of a “strong” AI application, but this is not yet possible. Finally, further studies with an adequate design (randomised clinical trials and/or prospective studies) are certainly necessary to confirm the results of our present work, and to validate this clinical protocol and the use of AI also with other implant systems, software and components. In fact, with different implant systems or connections, results may vary, and this should be adequately investigated.

## Conclusions

In this retrospective clinical study, a full digital protocol employing AI allowed the successful restoration of single locking-taper implants with MZCs cemented on customised hybrid abutments. In fact, 90 patients restored with 106 implant-supported MZCs were included in the study. The quality of the fabrication of individual hybrid abutments was high, as it revealed a mean deviation of 44 μm (± 6.3) between the original CAD design of the zirconia abutment, and the mesh of the zirconia abutment captured intraorally at the end of the provisionalization. At the delivery of the MZCs, the marginal adaptation, quality of interproximal and occlusal contacts, and aesthetic integration were excellent. During the period of observation and the follow-up, few biologic (1.9%) and prosthetic (5.7%) complications affected the implant-supported MZCs, for a three-year cumulative survival and success of 99.0 and 91.3%, respectively. Further studies with a longer follow-up and with different prosthetic restorations (such as implant-supported fixed partial prostheses) are needed to confirm the validity of this protocol.

## Data Availability

The complete documentation of all patients enrolled in this study belong to the authors, and are available only upon reasonable request.

## Abbreviations

3D: three-dimensional
AI: Artificial intelligence
CAD: Computer assisted design
CBCT: Cone beam computed tomography
CI: Confidence interval
ML: Machine learning
MZCs: Monolithic zirconia crowns
PMMA: Polymethylmethacrylate
SD: Standard deviation
STL: Stereolitographic
